# Impact of ^68^Ga-DOTATOC PET/CT in comparison to ^111^In-Octreotide SPECT/CT in management of neuro-endocrine tumors: A case report

**DOI:** 10.1097/MD.0000000000019162

**Published:** 2020-02-14

**Authors:** Anna Tolomeo, Gaetano Lopopolo, Vincenzo Dimiccoli, Luana Perioli, Sergio Modoni, Antonio Scilimati

**Affiliations:** a Radiopharmaceutical Division, ITEL Telecomunicazioni srl, Ruvo di Puglia; b Department of Pharmaceutical Sciences, University of Perugia, Perugia; c Nuclear Medicine Department, University Hospital “Ospedali Riuniti”, Foggia; d Department of Pharmacy-Pharmaceutical Sciences, University of Bari, Bari, Italy.

**Keywords:** ^111^In-Octreotide, ^18^F-FDG, ^68^Ga-DOTATOC, ileocecal-NET, neuroendocrine tumors, PET/CT

## Abstract

**Rationale:**

In the diagnostics of neuroendocrine tumors (NETs), scintigraphy and Single Photon Emission Computed Tomography/Computed Tomography (SPECT/CT) with ^111^Indium-Octreotide occupy a prominent place.

The introduction in clinical practice of ^68^Gallium-labelled somatostatin analogues (DOTA-TOC, DOTA-TATE, DOTA-NOC) for Positron Emission Tomography/Computed Tomography (PET/CT), significantly improved NETs diagnostics due to greater sensitivity and improved lesion detection in addition to better patient convenience and decreased radiation dose.

**Patient concerns:**

We report a case of a patient who was diagnosed with a neuroendocrine tumor of the ileocecal valve.

**Diagnoses:**

Diagnosis was made by ultrasonography, CT, and colonoscopy. Hystology after surgery was G2 NET of ileo-cecal valve. Restaging was carried out by ^111^In-Octreotide SPECT/CT and, 1 month later, by ^68^Ga-DOTATOC PET/CT. ^18^F-FDG PET/C was also carried out.

**Interventions:**

^68^Ga-DOTATOC PET/CT showed larger disease that modified disease management from surgery to medical treatment.

**Outcomes:**

After an initial improvement in the patient clinical condition, the tumor caused a worsening with the appearance of ascites.

**Lessons:**

^68^Ga-DOTA-conjugate PET/CT is appropriate in low and intermediate NET (Ki67 index respectively ≤3% and 3%–20%) characterized by better survival and better response after Peptide Receptor Radionuclide Therapy.

^18^F-FDG is mostly useful in high grade (G3) of disease, so that ^68^Ga-DOTA-conjugate SUV and ^18^F-FDG SUV have an opposite trend in relation to the tumor grade. ^68^Ga-DOTATOC PET/CT changes, as in our case, therapeutic management in about 40% of cases.

## 1 Introduction

Neuroendocrine tumors (NETs) originate from the widespread neuroendocrine system and, therefore, can arise in any body district. In two thirds of cases they can be born in the gastroenteropancreatic tract. In terms of frequency, NETs are usually considered rare neoplasms when compared to the corresponding non-neuroendocrine neoplasms.

The frequency of NETs is low, even if increasing in the last years (1–5 new cases/100,000 inhabitants / year), but the prevalence is high (35 cases/100,000 inhabitants) because most of them are well differentiated and slowly growing tumors with an indolent clinical course.^[1]^ In Italy, the overall incidence rate is 4.15%, with 2697 new cases in 2015, without significant differences between males and females, and reaches 11.27% in the age group over 65 years. The overall prevalence rate is 40.73%, with 23,937 estimated prevalent cases, with an average 5-year survival of approximately 75%. Small intestine and the bowel account for about a quarter of all cases (25.3%).^[2]^ NETs express a variable amount of 5 different somatostatin receptors (SSTR1-5) which have been used as targets for diagnostic radionuclide imaging and therapy.

Radiolabelled somatostatin, which could be the best probe, cannot be used because of its in vivo very short half-life (1–3 minutes); therefore, the octreotide derivate as somatostatin analogues endowed with a sufficient long half-life are employed. Scintigraphic detection was performed first by radioiodinated analogues^[3]^ followed by ^111^Indium (In)- and ^99m^Technetium(Tc)-radiolabelled analogues.^[4]^ Further technical improvements were achieved by the use of SPET/CT hybrid scanner^[5]^ which improved detection via a better target-to-background ratio and better anatomical localization due to CT images.

The subsequent availability of positron emitters radionuclides like ^68^Gallium (Ga) for PET/CT scan led to synthesis of radiolabelled analogues in which, the interposition of specific chelator agents such as di-ethylene-triaminepentaacetate (DTPA) or 1,4,7,10-tetraazacyclo-dodecane- 1,4,7,10-tetraacetic acid (DOTA) between somatostatin analogue and radionuclide made the link more stable.

PET/CT hybrid imaging with ^68^Ga-DOTA-conjugated (DOTA-TOC, DOTA-TATE and DOTA-NOC) overcomes, moreover, technical limitations of SPECT studies improving resolution and efficiency with more accurate images.

The amount of uptake, which reflects the receptors expression, is measured by a semi-quantitative index, the Standardized Uptake Value (SUV). SUV is the most used index in clinical PET/CT imaging, because it overcomes the differences in patients weight and size and among the amount of the injected dose of radiopharmaceuticals.

It has also a specific role in assessing disease behavior and response to different treatments. In ^68^Ga-DOTA-conjugates PET/CT, SUV reflects the amount of somatostatin receptors in neuroendocrine tumor.

Radiolabelling by beta or beta-gamma emitters radionuclides ^90^Yttrium (Y) or ^177^Lutetium (Lu) paved the way to Peptide Receptor Radionuclide Therapy (PRRT).^[6]^

Despite the different affinity of the 3 ^68^Ga-DOTA-conjugates towards somatostatin receptors,^[7]^ they have a comparable diagnostic value in the detection of NETs lesions, and nevertheless the approximately 10-fold higher affinity in binding SSTR2 of ^68^Ga-DOTA-TATE, which did not prove to be clinically relevant, SUVmax measurements with ^68^Ga-DOTA-TOC tended to be higher than their ^68^Ga-DOTA-TATE counterparts.^[8]^

^68^Ga-DOTA-conjugates proved to be superior to ^111^In-Octreotide SPECT/CT in NETs diagnostics, with a significant improvement in investigation time (90 minutes vs 24 hours), decreased radiation dose (3 mSv vs 9 mSv) and therefore, better patient convenience. In bowel NETs, moreover, the rate of positive lesions detected by ^68^Ga-DOTATATE PET/CT and ^111^In-Pentetreotide SPECT/CT was respectively 49/51 (96.1%) and 7/51 (13.7%) that led to change the management of 32.8% patients.^[9]^

^18^Fluoro-deoxy-glucose (F-FDG) PET/CT is also used in NETs diagnostics, but it is reserved to less differentiated and more aggressive tumors.^[10]^ In ^18^F-FDG PET/CT, SUV reflects increasing of glucose metabolism by cancer cells and consequently the tumor proliferative activity.

^18^F-FDG and ^68^Ga-DOTA-conjugates have therefore different indications and their SUV values have an opposite trend in relation to the tumor grade.^[11]^ In this report, we present a case of a patient with neuroendocrine tumor arisen on ileo-cecal valve who underwent a complete radionuclide diagnostic imaging beyond standard radiological imaging.

## 2 Case report

A 68-year-old man, due to anemia, performed ultrasound and abdominal CT which showed a voluminous 10-cm nodule in the right hepatic lobe and retropancreatic bulky disease imprinting the inferior vena cava at the renal confluence. Clear thickening of the walls of the last ileal loop was associated with involvement of the caecal fundus and of the root of the mesentery.

Colonoscopy showed an easily bleeding polylobed vegetative mass, which involved the ileocecal valve and almost completely obstructed the visceral lumen.

Laboratory tests showed chromogranin A levels > 50 nmol/L.

Biopsy and hystology results showed an ulcerated neuroendocrine tumor of the ileocecal valve with Ki67 index approximately 12% (G2 sec. WHO 2010) and hepatic metastasis.

Immunohistochemistry showed positivity for Cromogranin A, synaptophysin, NSE and CD56.

The tumor was therefore staged (TNM/AJCC) as pT4N1M1.

Patient underwent surgery with right hemicolectomy and right hepatectomy and subsequent treatment by “cold” somatostatin analogues.

**Figure 1:**
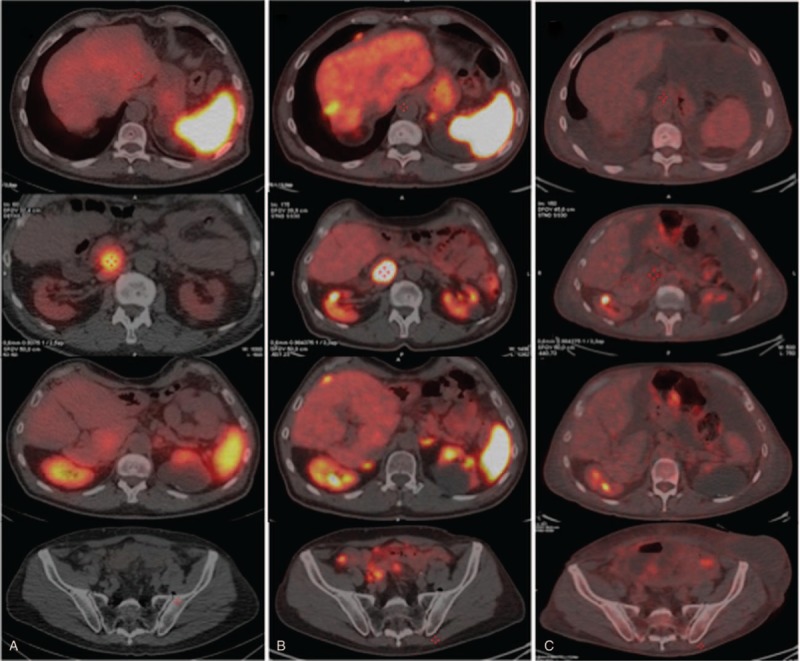
Restaging with ^111^In-Octreotide SPECT/CT scan (column a), ^68^Gallium-DOTATOC (column b), and ^18^F-FDG PET/CT scan (column c) of the patient, performed after colectomy and partial right hepatectomy and subsequent treatment by “cold” somatostatin. Since NET is a G2 tumor (Ki67 index 12%), positivity can observed in columns a and b. ^111^In-Octreotide SPECT-CT scan (column a) showed persistence of the retropancreatic uptake without other significant uptake areas. ^68^Ga-DOTATOC PET/CT scan (column b) confirmed uptake behind the pancreas head (SUV max 56.8) and showed multiple nodules of peritoneal implants in the periglissonian space, omentum, mesenteric region and pelvis (SUV max 13.8-15.4). ^18^F-FDG PET/CT scan (column c): metabolic activity in retropancreatic lesion was absent (SUV max 1.9) and mild pathological nodal uptake was observed in mesenteric region, with a conspicuous ascitic and pleural effusions.

One month after surgery, the patient underwent ^111^In-Octreotide SPECT-CT scan (118 MBq i.v.) which showed retropancreatic increased expression of somatostatin receptors without any other uptake areas (Fig. 1a).

The SPECT images were acquired on a dual-head gamma-camera (Discovery NM/CT 670, GE Healthcare, Little Chalfont, UK). Iterative method using ordered-subset expectation maximization (OSEM), with 2 iterations and 10 subsets, and Butterworth post-filter (0.48 critical frequency, power 10) were used for reconstruction procedure.

About a month later, the patient also underwent to ^68^Ga- DOTATOC PET/CT examination, while he was prepared for the surgical removal of the known retropancreatic mass. PET/CT scan was carried out by Discovery 710PET/CT (GE Healthcare, Little Chalfont, UK).

This radiopharmaceutical was synthesized in accordance with the European Pharmacopoeia (Gallium[^68^Ga] Edotreotide injection 01/2013: 2482 corrected 8.6) and 158 MBq were intravenously administrated to the patient.

**Figure 2:**
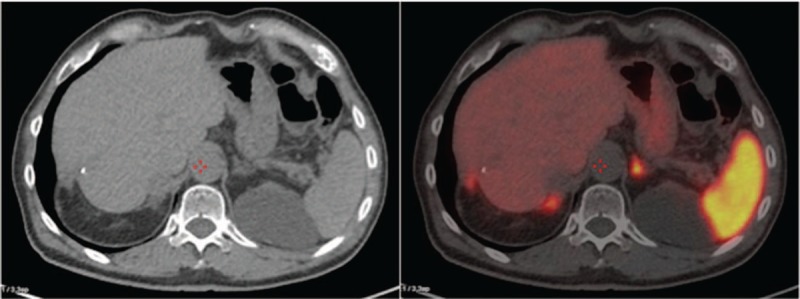
^68^Ga-DOTATOC PET/CT scan: as additional finding, in the medial lip of the left adrenal gland, surrounded by physiological uptake (SUV max 14.9), was found a nodular area suggesting adenoma (SUV max 27.5), confirmed by surgery.

Scan showed the known gross lymphnode mass in the right para-caval site, behind the head of the pancreas (SUV max 56.8) but also showed multiple nodular areas, as from peritoneal implants, in the peri-glissonian, omental, mesenteric and pelvic area (Fig. 1b). Moreover, inside the medial lip of the left adrenal gland, in the context of physiologic radiopharmaceutical uptake (SUV max 14.9), a nodular area compatible with adenoma was shown (SUV max 27.5) (Fig. 2).

These results modified the therapeutic course from surgical (curative secondary resection) to pharmacological treatment.

**Figure 3:**
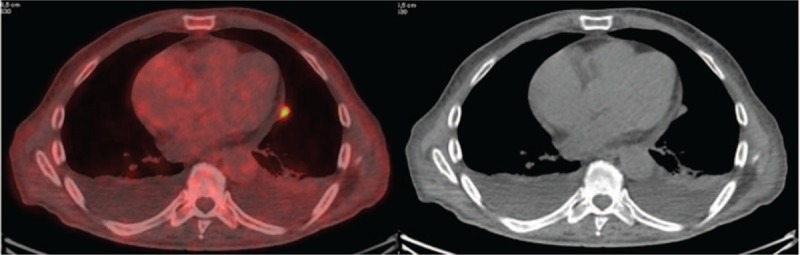
^18^F-FDG-PET/CT scan: small uptake in the left paracardiac area at the anterior segment of the lower lobe of the left lung not shown by ^68^Ga-DOTATOC PET/CT scan.

For completeness, 1 month later, we decided to carry out a ^18^F-FDG PET/CT scan (256 MBq i.v) (Fig. 1c) which showed a small but intense uptake (SUV max 7.8) in the left paracardiac area at the anterior segment of the lower lobe of the left lung, and uptake in some mesenteric nodes (Fig. 3).

**Figure 4:**
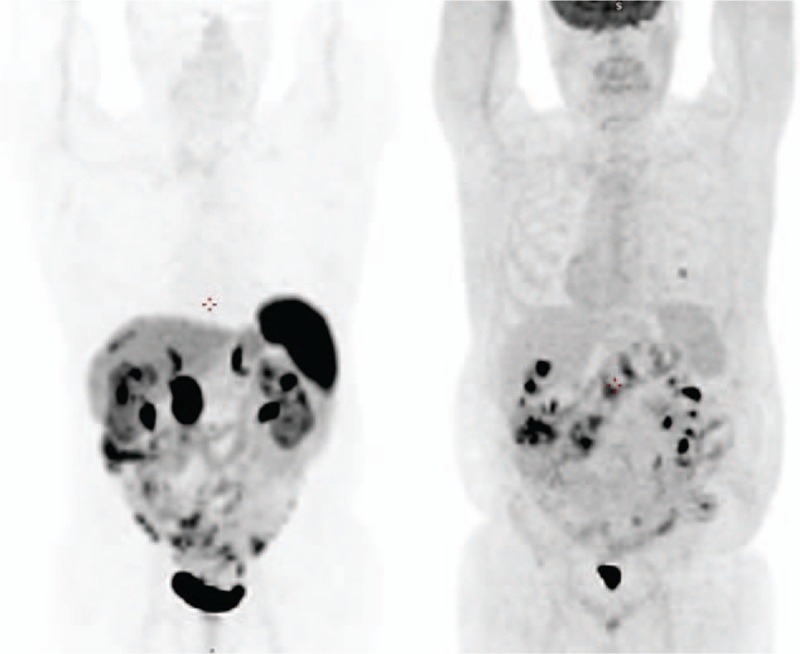
Maximum Intensity Projection (MIP) of ^68^Ga-DOTATOC PET/CT (left) and ^18^F-FDG PET/CT (right) showing the body physiological and pathological distribution of the 2 radiopharmaceuticals: the 2 images can be considered as complementary showing low and intermediate grade lesions (left image) and high grade lesions (right image) that can derive from tumor cell dedifferentiation or, as in the case of the pulmonary nodule, primary.

The Maximum Intensity Projection (MIP) images (Fig. 4), also known as volumetric images, summarize the physiological and pathological body distribution of the 2 PET radiopharmaceuticals (^68^Ga-DOTA-TOC on the left, ^18^F-FDG on the right).

## 3 Discussion

Neuroendocrine neoplasms (NEYs) are a heterogeneous group of tumors that originate from neuroendocrine cells and grow in a lot of different organs, mainly in the gastrointestinal tract and the lungs.

Most of NETs express somatostatin receptors (SSTR), which can be used as targets for radionuclide imaging and therapy.

^68^Ga-DOTA-conjugates PET/CT showed higher sensitivity than ^111^In-Octreotide scintigraphy (96.1% vs 13.7% in bowel NETs)^[9]^ due to its better intrinsic and physical resolution.

Another issue is the correct indication of use of these radiopharmaceuticals. For this purpose, it is very important to know the proliferative activity of the tumor, which is measured by the Ki67 index.

Radiolabelled somatostatin analogues are appropriate in NETs with low or intermediate proliferative activity grade. (Ki67 index respectively ≤3% and 3%–20%). Ki67 index well correlates with ^69^Ga-DOTA-conjugates uptake expressed by SUVmax, whereas ^18^F-FDG uptake is higher in tumors with high proliferative activity (Ki67 index >20%).^[11]^

^68^Ga-DOTA-conjugates SUVmax is, therefore, an important prognostic factor in G1 and G2 NETs because it well correlates with progression-free survival.^[12,13]^

Primary indications of radiolabelled somatostatin analogues are in diagnosis and staging, re-staging, monitoring response to treatments and more generally to determine the receptor status in order to adopt correct management decisions as well as patients selection for PRRT (with ^177^Lu or ^90^Y–DOTA-conjugates).

Secondary indication is to assess non-NETs with low or varying SSTR expression.^[7,14]^

PET/CT with ^68^Ga-DOTA-conjugates has a significant impact on general management which, in most of cases, about 40% in midgut NETs, was nonsurgical and more conservative.^[15]^

However, functional imaging is a high sensitive method and false-positive findings are always possible. These outcome triggers further tests with increased costs. Therefore, higher care must be taken to correlate the findings with the patient clinical conditions.^[16]^

The presented case as an excellent example of the diagnostic advantages of ^68^Ga-DOTA-TOC PET/CT in comparison with ^111^In-Octreotide SPECT/CT, and ^18^F-FDG PET/CT helps to highlight the various degrees of tumor differentiation during its spread. In the same patient we found also an adrenal adenoma. Even if adrenal medulla has a physiologic uptake of ^68^Ga-DOTA-conjugates, it is not rare to find, in course of PET/CT with these radiopharmaceuticals, other pathologic neuroendocrine tissue characterized by a higher SUVmax value^[17,18]^ such as adenoma suggesting possible undiagnosed familiar NET syndromes.

Finally, ^68^Ga-DOTA-conjugates uptake, besides being a favorable prognostic indicator, is also a good prognostic index of response to PRRT.^[19]^

### 3.1 Statement

Patient has provided written informed consent for publication of the case. The authors would like to thank the patient for authorizing the use the medical information contained in the present article.

## Author contributions

**Conceptualization:** Anna Tolomeo, Vincenzo Dimiccoli and Antonio Scilimati.

**Data curation:** Sergio Modoni.

**Formal analysis:** Sergio Modoni.

**Investigation:** Sergio Modoni.

**Methodology:** Sergio Modoni.

**Supervision:** Anna Tolomeo, Gaetano Lopolopo, Vincenzo Dimiccoli, Luana Perioli and Antonio Scilimati.

**Validation:** Gaetano Lopolopo, Vincenzo Dimiccoli, Luana Perioli and Antonio Scilimati.

**Writing – original draft:** Anna Tolomeo, Sergio Modoni.

**Writing – review & editing:** Anna Tolomeo, Gaetano Lopolopo, Vincenzo Dimiccoli, Luana Perioli and Antonio Scilimati.
